# Identifying Minimum Threshold Level of Trial Crossover Rates Yielding Nonsignificant Overall Survival Benefit Associated with Cancer Treatments: A Systematic Literature Review of HTA Submissions

**DOI:** 10.36469/001c.163160

**Published:** 2026-07-07

**Authors:** Anandaroop Dasgupta, Ankita Kaushik, Barinder Singh, Sumeet Attri, Billy Amzal, Oleg Gluz

**Affiliations:** 1 Gilead Sciences, Foster City, California, USA; 2 Pharmacoevidence, Mohali, India; 3 Phastar, Paris, France; 4 Breast Center Niederrhein, Evangelical Hospital Johanniter Bethesda, Moenchengladbach, Germany

**Keywords:** crossover rate, oncology trials, health technology assessment, intention‑to‑treat analysis, overall survival, treatment effect, statistical significance

## Abstract

**Background:**

Crossover distortion in oncology trials occurs when control-arm patients switch to agent(s) in the experimental arm, potentially attenuating treatment effects in intention-to-treat (ITT) overall survival (OS) analyses. This complicates health technology assessment (HTA) decision-making by masking true treatment benefits. Understanding crossover thresholds compromising ITT OS statistical significance is critical for interpreting trial outcomes.

**Objective:**

We aimed to identify minimum threshold beyond which crossover rates are associated with loss of ITT OS statistical significance in advanced/metastatic breast, colorectal, gastrointestinal stromal, lung, prostate, and renal cancers.

**Methods:**

A systematic literature review of HTA reports (2013-2024) from NICE-UK, CDA-Canada, HAS-France, G-BA/IQWiG-Germany, PBAC-Australia, AIFA-Italy, AEMPS-Spain, and ICER-US was conducted. Searches were augmented with information from clinical practice guidelines and product labels from regulatory agencies. Submissions reporting crossover rates, with/without crossover-mitigation strategies applied, were analyzed to quantify the relationship between crossover rates and ITT OS statistical significance.

**Results:**

Fifty unique trials from 114 submissions reported crossover rates (NICE = 25, HAS = 19, CDA = 15, PBAC = 13, G-BA/IQWiG = 32, AEMPS = 9, ICER = 1), with crossover-mitigation strategies applied in 26 trials. A hypothesis-generating minimum benchmark of crossover rate (the ratio of control-arm participants switching to agent(s) in the experimental arm) ~42.7% was identified above which ITT OS lost statistical significance. Trials with high crossover rates (42.7%-88%) showed nonsignificant OS improvements, whereas lower rates (<42.5%) demonstrated significant OS benefits. Higher crossover rates were observed in trials with built-in vs natural crossover (52.9% vs 8.1%). In first-line setting, crossover rates >42.5% typically diluted OS benefit. In later-line and refractory diseases, OS significance often persisted beyond 42.7% threshold due to limited confounding by post-progression treatment options and faster death event accrual.

**Conclusion:**

Crossover impact varied by tumor type, treatment line, crossover type, and trial maturity. Crossover >42.7% generally compromised ITT OS significance; however, OS maturity attained in the trial or sustained efficacy of the intervention beyond progression may preserve statistical significance despite high crossover. A crossover rate exceeding 42.7% was associated with nonsignificant ITT OS outcomes in most trials, particularly in first-line settings. OS benefits may persist in later-line settings with fewer post-progression treatment alternatives. This evidence supports contextual interpretation of crossover-affected OS during HTA decision-making.

## BACKGROUND

Treatment crossover in oncology trials represents a significant methodological challenge impacting interpretation of survival outcomes and subsequent health technology assessments (HTAs). Treatment crossover occurs when patients randomized to the control group switch to agent(s) in experimental arm during follow-up, typically after disease progression. This phenomenon is particularly common in cancer trials for ethical and practical reasons. Ethically, when interim analyses indicate a positive treatment effect, it may be deemed inappropriate to deny control group patients access to a potentially beneficial therapy, especially when no other nonpalliative treatments are available in the subsequent line of setting. Practically, allowing treatment crossover can facilitate patient recruitment and retention in clinical trials.^1^ This pioneering, active crossover design is welcomed by patients and healthcare professionals for its patient centric approach, allowing control arm patients to receive proven effective treatment as soon as possible. Furthermore, crossover in oncology studies can occur through more than one pathway. Built-in crossover (protocol-directed or crossover-by-design) is planned and follows explicit eligibility criteria, therefore allowing more predictable analytic adjustments. In contrast, natural crossover refers to situations in which patients gain access to the investigational treatment outside the study protocol because it becomes available in routine clinical care.

When treatment crossover occurs, the standard intention-to-treat (ITT) analysis, whereby data are analyzed according to the arms to which patients were randomized, becomes a biased approach for economic evaluations. While ITT analysis maintains randomization balance and provides a valid comparison of randomized groups, the survival benefit associated with the experimental treatment becomes diluted because control group patients who switch treatments may experience extended survival compared to what would have been observed had they remained on control treatment.^1,2^ This distortion particularly affects overall survival (OS) analyses, which are crucial for clinical effectiveness assessment and economic evaluations requiring a lifetime horizon perspective, leading to overestimation of the incremental cost-effectiveness ratio, and inappropriate resource allocation decisions.^1^

The importance of identifying a crossover rate benchmark, which when exceeded shows nonsignificant ITT OS results, relates directly to HTA decision-making. Payers are often insensitive to the impact of high crossover rates on OS, partly because trials are frequently designed to permit patients to receive effective therapies in subsequent lines of treatment. However, when treatment crossover occurs, observed trial results may not reflect the survival outcomes expected under standard clinical practice, creating uncertainty around the true treatment benefit and making it difficult for decision-makers to establish appropriate cost-effectiveness thresholds.^2^

This systematic literature review (SLR) aims to investigate the impact of treatment crossover on survival analyses in oncology trials and identify a hypothesis-generating benchmark associated with crossover rates for interpreting nonsignificant OS results in HTAs. Crossover rates were additionally examined by crossover type, blinding status, and line of therapy (LOT). By addressing these methodological challenges, the findings will contribute to more informed HTA decision-making in cancer care evaluations.

## METHODS

An SLR of published HTA reports from 8 major agencies (National Institute for Health and Care Excellence [NICE], Haute Autorité de Santé [HAS], Institute for Quality and Efficiency in Health Care/Federal Joint Committee [IQWiG/G-BA], Pharmaceutical Benefits Advisory Committee [PBAC], Institute for Clinical and Economic Review [ICER], Agencia Española de Medicamentos y Productos Sanitarios [AEMPS], Agenza Italiana del Farmaco [AIFA], and Canada’s Drugs Agency (CDA), was conducted between 2013-2024, by retrieving data from their websites. Search strategies were augmented with information from clinical practice guidelines and product labels from regulatory agencies. The study focused on advanced/metastatic tumors across multiple cancer types, including breast, colorectal, gastrointestinal stromal (GIST), non-small cell lung (NSCLC), prostate, and renal cell (RCC) cancers. Data on product, trial name, indication, crossover rates for the most updated follow-up, statistical significance of ITT OS, crossover type (built-in or natural), LOT, data maturity, and follow-up duration were systematically extracted and analyzed. Detailed inclusion/exclusion criteria, search strategy, selection, and data extraction process are provided in the **Online Supplementary Material**. Submissions were categorized by crossover rate and corresponding ITT OS outcomes to identify a hypothesis-generating benchmark at which crossover begins to compromise statistical significance. The threshold was initially determined by visual inspection and subsequently validated using the Maximally Selected Rank Statistics (Maxstat) method to identify the optimal cutpoint at which ITT OS lost significance.

## RESULTS

Screening of 114 product submissions to 8 HTA bodies (NICE [n = 25], HAS [n = 19], CDA [n = 15], PBAC [n = 13], G-BA/IQWiG [n = 32], AEMPS [n = 9], ICER [n = 1], AIFA [n = 0]) identified 50 unique clinical trials reporting crossover rates (see Supplementary Online Material for PRISMA diagram). Crossover-mitigation strategies were applied in 26 trials (53 submissions) and not applied in 24 trials (61 submissions).

**Figure 1.**
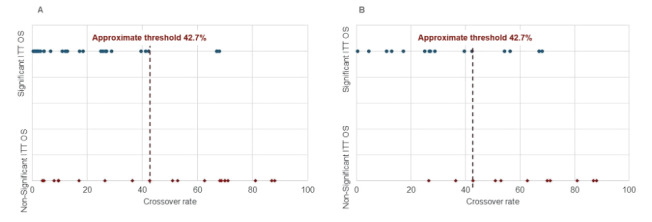
Relationship Between Crossover Rate and ITT OS Statistical Significance Across (**A**) 50 Trials^a^ and (**B**) 26 Trials^b^ Each data point represents an individual trial plotted by control-arm crossover rate (x-axis, %) against ITT OS statistical significance (y-axis: significant or nonsignificant). The vertical red dashed line denotes the approximate crossover threshold beyond which ITT OS significance is predominantly lost: 42.7% in both panel (A) and panel (B). In both panel (A) and (B), trials are distinguished by outcome: blue circles = significant ITT OS; red diamonds = nonsignificant ITT OS, visually highlighting the divergence in significance patterns above and below the threshold. Abbreviations: ITT, intention-to-treat; OS, overall survival. ^a^Regardless of application of crossover-mitigation strategies (n = 50). ^b^With application of crossover-mitigation strategies (n = 26).

The relationship between crossover rates and ITT OS statistical significance revealed a clear pattern across trials (**Figure 1**). Trials with low crossover rates (<42.5%) predominantly demonstrated significant ITT OS results (75.8% of 33 trials; hazard ratio [HR] range, 0.56^3^-0.80^4^), while those with high crossover rates (>67.5%) universally showed nonsignificant results (all 8 trials: HR range, 0.81^5^-1.32^6^). Visual inspection identified an approximate threshold of 42.7% crossover rate across past oncology trials (crossover rate <42.7%: median HR, 0.72; range 0.56^3^-1.01^7^; crossover rate ≥42.7%: median HR, 0.87; range, 0.41^8^-1.32^6^), beyond which ITT OS results frequently became nonsignificant (**Figure 1**). The Maxstat-derived optimal cutpoint of 42.4% was a statistically significant (*P* = .015) discriminator between clinical trials with crossover rates yielding statistically significant and nonsignificant OS outcomes. This optimal cutoff point was similar to the visually derived threshold of 42.7%. Overall, 74% of 50 trials were consistent with the 42.7% threshold: 75.7% of 33 trials with a crossover rate <42.7% reported statistically significant ITT OS, while 70.6% of 17 trials with a crossover rate ≥42.7% reported ITT OS nonsignificant. The crossover rate threshold remains the same following the exclusion of 13 outlier trials. Furthermore, the median crossover rate was higher among trials with statistically nonsignificant ITT OS (median 52.0%, range 3.8%^9^-88.0%^10^) vs statistically significant ITT OS (median, 20.0%; range, 0.4%^11^-68.0%^8^) (see **Online Supplementary Material**).

This relationship between crossover rates and statistical significance is particularly evident across different cancer types and treatment scenarios (**Table 1**). When analyzing more mature OS data (later or most recent data cutoffs), trials with crossover rates exceeding 50% typically showed nonsignificant ITT OS results. The empirical threshold identified was approximately 39%-40% crossover, above which statistical significance was generally lost despite data maturity. For trials with early data cutoffs or immature OS, the threshold for maintaining statistical significance was lower. With immature data, crossover rates above 42-43% typically resulted in nonsignificant ITT OS. Further, treatments with substantial clinical efficacy, such as pembrolizumab (KEYNOTE-024: HR, 0.60; 95% confidence interval [CI], 0.41-0.89^12^; KEYNOTE-189: HR, 0.49; 95% CI, 0.38-0.64),^13^ cemiplimab (EMPOWER-LUNG-1: HR, 0.67; 95% CI, 0.52-0.87),^14^ and apalutamide (TITAN: HR, 0.65; 95% CI, 0.53-0.79),^15^ could sustain statistical significance even with crossover rates of 39%-56%.

**Table 1.** Relationship Between Crossover Rates and Statistical Significance of ITT OS Results

**Crossover Rate, %**	**ITT OS Statistical Significance**	**Representative Trials (Crossover Rate, %)**	**Cancer Types**	**HTA Bodies**
0-3	All significant	STUDY 301 (0.4), CheckMate-057 (0.7), METEOR (2.10), CORRECT (1.57), MAGNITUDE (2.3), IMpower150 (1.5), KEYNOTE-355 (2.1), CheckMate-214 (3.0), VISION (1.1)	mBC, nonsquamous mNSCLC, clear cell mRCC, BRCA 1/2 mutant mCRPC, ECOG 0-1 mCRC, EGFR+/ALK+ mNSCLC, mTNBC, intermediate/poor risk, advanced mRCC, PSMA +ve mCRPC	NICE, CDA, PBAC, G-BA/IQWiG
>3-30	Predominantly significant	HER2CLIMB (12.9), EMILIA (27), CLEOPATRA (11), DESTINY-BREAST-03 (25), CodeBreak-200 (26.4)*, ARCHES (28.8), COU-AA-302 (17.2), KEYNOTE-407 (26.7), PREVAIL (4.4), JMEN (18.50), DESTINY-Breast02 (25.7), LATITUDE (12), PALOMA-3 (17.0)*, PALOMA-2 (8.1)*, CABOSUN (3.8)*, ALEX (9.5)*, LUX-Lung 3 (9.6)*, TROPiCS-02 (6.7), CheckMate-017 (4.4), RELAY (4.4)*	HER2+ mBC, HR+/HER2- mBC, mNSCLC with KRAS-G12C mutation, mHSPC, mCRPC, squamous mNSCLC, nonsquamous mNSCLC, mPC, mBC, intermediate/poor risk advanced mRCC, EGFR+ mNSCLC, ALK+ mNSCLC, high-risk mHSPC	NICE, HAS, CDA, PBAC, G-BA/IQWiG, ICER
30-42.5	Mostly significant	TITAN (39.5), EMPOWER-LUNG-1 (42.4), FLAURA (41.3), KEYNOTE-177 (36.4)*	mHSPC, PD-L1+ (TPS ≥50%) mNSCLC, EGFR+ mNSCLC, MSI-H/dMMR mCRC	NICE, CDA, PBAC, AEMPS
>42.5-55	Mostly nonsignificant	ASCEND-4 (42.7), ALTA-1L (52.9), VEG105192 (51.0), KEYNOTE-024 (54.3)**	ALK+ mNSCLC, mRCC, PD-L1+ (TPS ≥50%) mNSCLC	NICE, AEMPS
>55-67.5	Mostly significant	KEYNOTE-189 (56.3), TIVO-1 (62.6)*, PROfound (67.0), IMPACT/D9901/D9902A (67.0)	EGFR/ALK-ve mNSCLC, r/mRCC, mCRPC, asymptomatic/minimally symptomatic (nonvisceral) mCRPC	HAS, NICE, CDA, G-BA/IQWiG, AEMPS
>67.5-70	Predominantly nonsignificant	INVICTUS (65.1)**, ASCEND-5 (68.1), ALUR (68.6), PROFILE1014 (70.0), LAURA (69.9)	Advanced GIST, ALK+ve mNSCLC, mCRPC, EGFR+ve mNSCLC	NICE, HAS, G-BA/IQWiG, AEMPS
>70	All nonsignificant	GRID (85), AURA3 (71.0), PROFILE1007 (87.0), RECORD-1 (81.0)	mGIST, EGFR and T790M mutation +ve NSCLC, ALK+ mNSCLC, mRCC	NICE, AEMPS

Trials are grouped into 6 crossover rate bands reflecting distinct patterns of ITT OS statistical significance, drawn from 50 unique clinical trials reported in the health technology assessment (HTA) reports (2013–2024) from 8 agencies: NICE (UK), CDA (Canada), HAS (France), G-BA/IQWiG (Germany), PBAC (Australia), AIFA (Italy), AEMPS (Spain), and ICER (US).

Abbreviations: ALK+, anaplastic lymphoma kinase positive; BRCA1/2, BReast CAncer gene 1/2; CDA, Canada’s Drug Agency; CRPC, castration resistant prostate cancer; EGFR, epidermal growth factor; GIST, gastrointestinal stromal tumor; HAS, Haute Autorité de Santé; HER2+, human epidermal growth receptor 2 positive; HSPC, hormone sensitive prostate cancer; HTA, health technology assessment; IQWiG/G-BA, Federal Joint Committee/Institute for Quality and Efficiency in Health Care; ITT, Intention-to-treat; KRAS G12C, Kirsten rat sarcoma gene with a glycine-to-cystein mutation at position 12 mBC, metastatic breast cancer; mNSCLC, metastatic nonsmall cell lung cancer; MSI-H/dMMR, microsatellite instability-high/deficient mismatch repair; NICE, National Institute for Health and Care Excellence; OS, overall survival; PBAC, Pharmaceutical Benefits Advisory Committee; PD-L1, programmed death ligand 1; PSMA, prostate-specific membrane antigen; RCC, renal cell carcinoma TPS, tumor proportion score.

Opposite trend: *Nonsignificant/similar; **statistically significant.

Predominantly: 70%-85% trials either reported statistically significant or nonsignificant.

Mostly: >50% of the trials either reported statistically significant or nonsignificant.

**Table 2** provides a comprehensive breakdown of crossover rate and statistical significance by LOT. The impact of crossover on ITT OS significance varied across lines of therapy. In first-line settings, crossover rates exceeding 42.5% generally dilute the OS benefit. This was evident in multiple first-line trials for metastatic NSCLC, where crossover rates of 42.7-70% consistently resulted in nonsignificant ITT OS (ASCEND-4,^16^ ALTA-1L,^17^ PROFILE 1014^18^). Similarly, in first-line metastatic RCC, crossover rates of 51% and 62.6% in the VEG105192^19^ and TIVO-1^20^ trials, respectively, resulted in similar rather than superior OS outcomes. In contrast, later-line and refractory disease settings demonstrated greater tolerance for higher crossover rates while maintaining statistical significance due to limited treatment options after progression and faster event accrual. The PROfound trial maintained statistical significance despite a 67% crossover rate, likely due to its extended follow-up of approximately 21 months.^21^ Similarly, the INVICTUS trial in fourth-line or further-line setting (4L+) showed significant OS despite 65.1% crossover.^8^ The analysis also identified exceptions where significant ITT OS was maintained despite high crossover in first-line settings, particularly the pembrolizumab KEYNOTE-024 and KEYNOTE-189 trials, with crossover rates of 54%^12^ and 56%,^13^ respectively. As noted in the NICE assessment, crossover was allowed with pembrolizumab being the standard treatment after progression to chemotherapy. However, the prolongation of survival could be recognized considering the sustained clinical benefit (beyond progression) associated with pembrolizumab in the intervention arm. This demonstrates that while the 42.7% threshold generally holds across settings, the clinical context and data maturity can significantly influence the outcome. Further, built-in crossover is associated with higher crossover rates (median, 52.90%; range, 0.40%^11^-88%^10^), and 52.2% of 23 trials exceeded the 42.7% OS significance threshold. However, natural crossover tends to be associated with lower crossover rates (median, 8.10%; range, 0.70%^22^-68.10%^6^), and only 2 of 25 trials exceed the 42.7% threshold (VEG105192,^19^ ASCEND-5^6^). Open-label trials are associated with higher crossover rates (median, 26.10%; range, 0.40%^11^-87.00%^23^) compared with double-blind trials (median, 17.9%; range, 1.60%^24^-88.00%^10^) (see **Online Supplementary Material**). The median crossover rates in trials where crossover eligibility was ascertained after unblinding and when interim analysis met statistical significance were 34.10% (range, 1.60%^24^-88.00%^10^) and 27.90% (range, 3.00%^25^-39.50%^15^), respectively.

**Table 2.** Crossover Rates and Statistical Significance of ITT OS Results by Line of Therapy

**Line of Therapy**	**Trial Name**	**Disease**	**Crossover Rate, %**	**ITT OS Significance**	**Type of Crossover**
1L	ALTA-1L	ALK+, mNSCLC	52.90	Nonsignificant	Built-in (protocol specified)
1L	PROFILE 1014	ALK+, mNSCLC	70.00	Nonsignificant	Built-in (protocol specified)
1L	ASCEND-4	ALK+, mNSCLC	42.70	Nonsignificant	Built-in (protocol specified)
1L	VEG105192	mRCC	51.00	Nonsignificant	Natural (protocol unspecified)
1L	TIVO-1	mRCC	62.60	Nonsignificant	Built-in (protocol specified)
1L	KEYNOTE-177	MSI-H/dMMR mCRC	36.40	Nonsignificant	Built-in (protocol specified)
1L	KEYNOTE-024^a^	PD-L1+, mNSCLC	54.30	Significant**	Built-in (protocol specified)
1L	KEYNOTE-189^b^	EGFR-, ALK- mNSCLC	56.30	Significant**	Built-in (protocol specified)
1L	EMPOWER-LUNG-1^b^	PD-L1+ (≥50%) mNSCLC	42.40	Significant	Built-in (protocol specified)
1L	TITAN	mHSPC	39.50	Significant	Built-in (protocol specified)
1L	CLEOPATRA	HER2+, mBC	11.00	Significant	Natural (protocol unspecified)
1L	KEYNOTE-407	mNSCLC, SQ	26.70	Significant	Built-in (protocol specified)
1L	PREVAIL	mPC	4.40	Significant	Built-in (protocol specified)
1L	ARCHES	mHSPC	28.80	Significant	Natural (protocol unspecified)
1L	COU-AA-302	mCRPC, asymptomatic	17.20	Significant	Natural (protocol unspecified)
1L	LATITUDE	High-risk mHSPC	12.00	Significant	Built-in (protocol specified)
1L	FLAURA	EGFR+ mNSCLC	41.30	Significant	Built-in (protocol specified)
1L	IMPACT^c^ D9901 D9902A	Asymptomatic/minimally symptomatic (nonvisceral) mCRPC	67.00	Significant**	Built-in (protocol specified)
1L	CheckMate-214	Intermediate/poor risk, advanced mRCC	3.00	Significant	Natural (protocol unspecified)
1L	IMpower150	NSQ mNSCLC; EGFR+/ALK+ mNSCLC	1.50	Significant	Natural (protocol unspecified)
1L	KEYNOTE-355	mTNBC PD-L1 (CPS ≥10)	2.10	Significant	Natural (protocol unspecified)
1L	CABOSUN	Intermediate/poor risk, locally advanced mRCC	3.80	Nonsignificant	Natural (protocol unspecified)
1L	ALEX	ALK+ NSCLC	9.50	Nonsignificant	Natural (protocol unspecified)
1L	LUX-Lung 3	EGFR+ mNSCLC	7.8-9.6	Nonsignificant	Natural (protocol unspecified)
1L	PALOMA-2	Postemenopausal HR+/HER2- mBC	8.10	Nonsignificant	Natural (protocol unspecified
1L	RELAY	EGFR+ mNSCLC	4.40	Nonsignificant	Natural (protocol unspecified
1L maintenance	JMEN	mNSCLC, NSQ	18.50	Significant	Natural (protocol unspecified)
2L	PROFILE1007	ALK+ mNSCLC	87.0	Nonsignificant	Built-in (protocol specified)
2L	RECORD-1	mRCC	81.0	Nonsignificant	Built-in (protocol specified)
2L	AURA3	EGFR T790M+ mNSCLC	67.10	Nonsignificant	Built-in (protocol specified - after amendment)
2L	ASCEND-5	ALK+ mNSCLC	68.10	Nonsignificant	Natural (protocol unspecified)
2L	LAURA	EGFR+ mNSCLC	69.90	Nonsignificant	Built-in (protocol specified)
2L	EMILIA	HER2+, mBC	27.00	Significant	Built-in (protocol specified - after amendment)
2L	Study301	Metastatic/advanced BC	0.40	Significant	Built-in (protocol specified)
2L	CheckMate-057	NSQ mNSCLC	0.70	Significant	Natural (protocol unspecified)
2L	CheckMate 017	SQ mNSCLC	4.40	Significant	Natural (protocol unspecified)
2L	PALOMA-3	HR +/HER2 – mBC	17.00	Nonsignificant	Natural (protocol unspecified
2L+	CodeBreak-200	KRAS-G12C mutation NSCLC	26.40	Nonsignificant	Built-in (protocol specified - after amendment)
2L+	DESTINY-BREAST-03	HER2+ mBC	25.00	Significant	Natural (protocol unspecified)
2L+	PROfound^d^	mCRPC, BRCA1/2	67.00	Significant**	Built-in (protocol specified)
2L+	METEOR	clear cell mRCC	2.10	Significant	Natural (protocol unspecified)
2L+	MAGNITUDE	BRCA 1/2 mutant mCRPC	2.30	Significant	Natural (protocol unspecified)
3L	GRID	mGIST	88.00	Nonsignificant	Built-in (protocol specified)
3L	ALUR	ALK+ NSCLC	68.60	Nonsignificant	Built-in (protocol specified)
3L	VISION	PSMA +, mCRPC	1.10	Significant	Natural (protocol unspecified
3L+	HER2CLIMB	HER2+, mBC	12.90	Significant	Natural (protocol unspecified)
3L+	TROPiCS-02	HR +, HER2 − mBC	6.70	Significant	Natural (protocol unspecified)
3L+	DESTINY-Breast02	HER2+ mBC	25.70	Significant	Natural (protocol unspecified)
3L+	CORRECT	ECOG 0-1 mCRC	1.57	Significant	Natural (protocol unspecified)
4L+	INVICTUS^e^	Advanced GIST	68.00	Significant**	Built-in (protocol specified - after amendment)

This table presents the control-arm crossover rates, ITT OS statistical significance, and crossover type (Built-in or natural crossover) reported among 50 oncology trials stratified by line of therapy (1L to 4L+). Built-in (protocol specified) is defined as crossover pre-specified in the trial protocol; built-in (protocol specified - after amendment) is defined as crossover was permitted following a protocol amendment; natural (protocol unspecified) is defined as crossover occurred outside protocol specification.

Abbreviations: 1L, first-line; 2L, second-line; 3L, third-line; 4L, fourth-line; ALK, anaplastic lymphoma kinase; BC, breast cancer; BRCA1/2, BReast CAncer gene 1/2; CPS, combined positive score; CRC, colorectal cancer; CRPC, castration-resistant prostate cancer; dMMR, deficient mismatch repair; ECOG, Eastern Cooperative Oncology Group; EGFR, epidermal growth factor receptor; GIST, gastrointestinal stromal tumor; HER2, human epidermal growth factor receptor 2; HR, hormone receptor; HSPC, hormone-sensitive prostate cancer; ITT, intention-to-treat; KRAS-G12C, Kirsten rat sarcoma viral oncogene homolog G12C; m, metastatic; MSI-H, microsatellite instability-high; NSCLC, non-small cell lung cancer; NSQ, non-squamous; OS, overall survival; PC, prostate cancer; PD-L1, programmed death-ligand 1; PSMA, prostate-specific membrane antigen; RCC, renal cell carcinoma; SQ, squamous; T790M, threonine-to-methionine substitution at codon 790 of EGFR; TNBC, triple-negative breast cancer

^a^Longer follow-up: 25.2 months, and pembrolizumab, as a second-line therapy, represents an approved and adequate component of the control group’s treatment strategy.

^b^Substantial clinical efficacy (Follow-up: 10.5 months and ~14 months in KEYNOTE-189 and EMPOWER-LUNG-1 trial, respectively).

^c^Mature data (follow-up not reported).

^d^Mature data and longer follow-up (21.9 months).

^e^Mature data and longer follow-up (25.3 months).

Opposite trend: **Statistically significant.

Further, same crossover rate threshold was yielded by restricting to 26 trials applying crossover-mitigation strategies (**Figure 1**). The findings were consistent with the full dataset, demonstrating higher median crossover rates in trials with statistically nonsignificant ITT OS (62.6% vs 26.7% in statistically significant trials), built-in crossover (47.8% vs 21.1% in natural crossover), open-label study design (42.7% vs 28.80% in double-blind trials), and second-line or later setting (67.00% vs 39.50% in first-line setting) (see **Online Supplementary Material**).

## DISCUSSION

Treatment crossover in oncology clinical trials represents a significant methodological challenge with profound implications for HTA and clinical decision-making. The analytical framework compared submissions with varying crossover rates against their ITT OS significance status, while controlling for potential confounding factors, including data maturity, LOT, blinding, follow-up duration, and magnitude of clinical benefit. This multiregional SLR revealed a hypothesis-generating benchmark of approximately 42.7% crossover rate, derived using visual inspection, above which statistical significance in ITT OS analyses is frequently compromised. Validation via the Maxstat method yielded a similar optimal cutpoint (42.4% vs 42.7%). These findings have substantial implications for interpreting clinical trial data and making healthcare policy decisions. As a robustness check, analysis restricted to 26 trials applying crossover-mitigation strategies yielded qualitatively similar conclusions.

When control arm patients switch to agent(s) in experimental arm, the true treatment effect becomes diluted in ITT analyses, a phenomenon termed “crossover distortion”, which affects precision and statistical significance, introduces bias and undermines OS estimate accuracy. This poses challenges for HTA bodies, which rely heavily on ITT OS data as the gold-standard endpoint for evaluating clinical efficacy. Trials with crossover rates exceeding 42.7% benchmark frequently report nonsignificant ITT OS results, despite potential treatment benefits observed in other endpoints. Higher crossover rates were more common in open-label vs double-blind designs. Crossover allowed at a later time (eg, after unblinding or when interim analysis met significance) yielded low rates in double-blind trials. However, we acknowledge that, from an ethical perspective, allowing later crossover is not a feasible option in open-label trials, particularly in cancer populations with high unmet needs.

Our study revealed important exceptions to this hypothesis-generating benchmark rule. Several trials maintained statistically significant OS improvements despite high crossover rates, particularly when supported by mature follow-up data. These exceptions highlight that while a 42.7% threshold provides a useful benchmark, other factors, including data maturity and magnitude of clinical benefit, can offset crossover-related OS dilution. Moreover, in therapy-rich first-line settings (eg, NSCLC), crossover above ~42.5% frequently dilutes ITT OS to nonsignificance. In contrast, in late-line refractory diseases (eg, 4L+ GIST), OS may remain significant even with >60% crossover, with limited follow-up maturity and few post-progression treatment options being key modifiers. For HTA bodies, these findings suggest current reliance on ITT OS as the definitive endpoint may need reconsideration when substantial crossover occurs. Also, HTA bodies may benefit from more flexible evidence assessment approaches when crossover exceeds ~42.7%, potentially assigning greater weight to progression-free survival or adjusted OS analyses (prespecified and post-hoc). Stratification by crossover mechanism further clarified this pattern: protocol-directed (built-in) crossover was associated with higher switching intensity, with 52.2% of built-in trials exceeding ~42.7% threshold for ITT OS significance, whereas among trials with natural crossover, only 2 studies crossed this threshold. This highlights that crossover-by-design elevates switching and thereby increases the risk of diluting OS effects under ITT.

Evidence from multiple HTA submissions across 8 HTA bodies indicates that these agencies have occasionally accepted significant treatment effects despite high crossover, when supported by mature data (n = 3)^8,21,26^ or substantial clinical efficacy (n = 3).^12–14^ For clinical trial design, these findings suggest several considerations. First, trial protocols should carefully consider crossover provisions, recognizing that rates exceeding 40%-43% may compromise the ability to demonstrate OS benefits. Second, when crossover is ethically necessary, trials should plan for longer follow-up periods to achieve data maturity, which may mitigate crossover effects on ITT OS. Third, statistical analysis plans should incorporate pre-specified adjustment methods to complement the primary ITT approach.

The strengths of the threshold identification approach include its empirical foundation based on HTA submissions and its practical applicability across multiple tumor types. When comparing these findings with previous research, this work extends our understanding by quantifying a pragmatic crossover threshold, whereas earlier studies primarily highlighted crossover as a general challenge without defining when it becomes critically problematic.^27–30^ However, this study has some limitations. The proposed 42.7% crossover rate benchmark was derived from descriptive patterns across heterogeneous trials rather than a definitive/universal cutoff. Multiple factors beyond crossover rate, including treatment effect size, sample size, follow-up duration, data maturity, trial design, tumor type, LOT, and treatment mechanism, may independently influence ITT OS significance and were not formally quantified. Although the threshold remained consistent following exclusion of the 13 outlier studies, meaningful heterogeneity across included trials suggests it is likely context-dependent rather than universal. Multivariable and subgroup analyses were not explored in this payer-focused descriptive study. A broader trial-level SLR followed by in-depth meta-analysis may provide a more robust and context-specific evaluation of the threshold in future research. Also, our research applies primarily to the HTA/countries included, and extrapolation to other geographies and jurisdictions would be speculative without further evaluation.

For stakeholders, these findings offer practical guidance. Regulators could consider the 40%-43% hypothesis-generating benchmark when evaluating trial evidence and seek crossover mitigation approaches to strengthen the evidence around lack of OS. Payers and HTA bodies might implement similar evidentiary standards while evaluating treatments with high crossover rates, particularly when supported by mature data.

## CONCLUSION

A descriptive trend was observed across oncology trials with treatment crossover, whereby a crossover rate exceeding 42.7% was associated with nonsignificant ITT OS outcomes in most trials. ITT OS significance in first-line trials was not observed beyond this hypothesis-generating benchmark. However, in later-line settings with fewer post-progression treatment alternatives, OS benefits may persist. Built-in crossover more frequently exceeded this benchmark than natural crossover, underscoring both the analytic challenge of crossover-by-design, and the need for prespecified/post-hoc adjustment and adequate patient follow-up for OS analysis. Such evidence supports contextual interpretation of crossover-affected OS, considering trial design, crossover timing and LOT. Considering how crossover impedes our understanding of an intervention’s benefit in prolonging OS, HTA bodies should allow supplemental evidence beyond clinical trials to support a treatment’s value story during reimbursement decision-making.

### Disclosures

A.D. and A.K. are employees of Gilead Sciences, Inc., and hold stock in the company. B.S. and S.A. are employees of Pharmacoevidence, who provided support in data collection and editing, which was funded by Gilead Sciences, Inc. B.A. is affiliated with Phastar, Paris, France, and provided scientific advice on the project. O.G. is affiliated with Breast Center Niederrhein, Evangelical Hospital Johanniter Bethesda, Moenchengladbach, Germany, and provided scientific advice on the project.

## Supplementary Material

Online Supplementary Material
