# Supplementary material for: Identifying Minimum Threshold Level of Trial Crossover Rates Yielding Nonsignificant Overall Survival Benefit Associated with Cancer Treatments: A Systematic Literature Review of HTA Submissions
Source: J Health Econ Outcomes Res. 2026 Jul 7;13(2):1–7. doi: 10.36469/001c.163160 (PMC13349023; doi:10.36469/001c.163160)
Supplement: Online Supplementary Material [file jheor_2026_13_2_163160_352795.pdf]

Journal of Health Economics and Outcomes Research

Online Supplementary Material

Identifying Minimum Threshold Level of Trial Crossover Rates Yielding Nonsignificant Overall Survival Benefit Associated with Cancer Treatments: A Systematic Literature Review of HTA Submissions.
JHEOR. 2026;13(2):1-7. doi:10.36469/jheor.2026.163160

Table S1: Eligibility Criteria 2

Table S2: HTA Search Strategy 4

Table S3: Characteristics of 50 Included Clinical Trials 7

Figure S1: PRISMA Flow Diagram 15

Figure S2: Box Plot of the Crossover Rate by Statistical Significance Across (A) All Trials Regardless of Application of Crossover Mitigation Strategies, (B) Trials with Application of Crossover Mitigation Strategies 16

Figure S3: Box Plot of the Crossover Rate by Line of Therapy Across (A) All Trials Regardless of Application of Crossover Mitigation Strategies, (B) Trials with Application of Crossover Mitigation Strategies 17

Figure S4: Box Plot of the Crossover Rate by Type of Crossover Across (A) All Trials Regardless of Application of Crossover Mitigation Strategies, (B) Trials with Application of Crossover Mitigation Strategies 17

Figure S5: Box Plot of the Crossover Rate by Blinding Status Across (A) All Trials Regardless of Application of Crossover Mitigation Strategies, (B) Trials with Application of Crossover Mitigation Strategies 18

This supplementary material has been provided by the authors to give readers additional information about their work.

This is an open-access article distributed under the terms of the Creative Commons Attribution 4.0 International License (CCBY-4.0). View this license's legal deed at http://creativecommons.org/licenses/by/4.0 and legal code at http://creativecommons.org/licenses/by/4.0/legalcode for more information.

Eligibility Criteria, Search Strategy, Selection Process, and Data Extraction

The review question was structured using the PICO framework, encompassing adult patients (≥18 years) with NSCLC, advanced/metastatic breast cancer, colorectal cancer, gastrointestinal stromal tumor (GIST), prostate cancer, and renal cell carcinoma (RCC), across any line of therapy. Interventions and comparators comprised systemic anticancer treatments, with outcomes of interest including product, trial name, indication, crossover rates, statistical significance of ITT OS, type of crossover (built-in or natural), line of therapy, data maturity, and follow-up duration. Eligible study designs included RCTs with preplanned or spontaneous treatment crossover. Detailed eligibility criteria are summarized in **Table S1**.

Search strategies were tailored to each HTA body's website. Bodies permitting disease-based searches (AEMPS, CDA/CADTH, ICER, IQWiG, and NICE) were searched using predefined cancer-specific keywords, while those permitting treatment-based searches (AIFA, FDA ODAC, HAS, and PBAC) were searched using a preestablished drug list derived from crossover trials identified in priority HTA reports. All drugs were searched irrespective of reimbursement status, with a date restriction of 2013-2024. Searches for AEMPS, AIFA, HAS, and IQWiG were conducted in their respective native languages (**Table S2**).

Screening was conducted in two stages. In the first stage, the titles were first assessed for eligibility by one reviewer against predefined criteria, with relevant titles retrieved for full-text review. In the second stage, full-text reports were evaluated to confirm eligibility and crossover design, informing the final inclusion or exclusion decision.

The relevant data from eligible submissions were extracted into structured Microsoft Excel templates by one reviewer and independently validated by a second reviewer for accuracy and consistency. Discrepancies were resolved through discussion, with a third reviewer consulted where necessary. Extracted variables included product name, trial name, indication, cancer type, patient population, intervention, comparator, crossover rate, type of crossover (built-in or natural), statistical significance of OS in the ITT analysis, line of therapy, data maturity, and follow-up duration. Where the same index RCT was submitted to more than one HTA body, the data was extracted from the submission providing the most comprehensive data.

Table S1. Eligibility Criteria

Criteria	Inclusion	Exclusion
Cancer type assessed	Locally advanced and/or metastatic:<br>• Breast cancer<br>• Non-small cell lung cancer<br>• Colorectal cancer<br>• Gastrointestinal stromal tumor<br>• Renal cell carcinoma<br>• Prostate cancer	• Early-stage or locally advanced tumors<br>• Tumors other than breast cancer, non-small cell lung cancer, colorectal cancer, gastrointestinal stromal tumor, renal cell carcinoma, and prostate cancer<br>• Enrolling a mix of cancer types or stages, where at least 80% of patients are not of interest for this review
Interventions assessed	Systemic anticancer treatments	• Treating non-systemic anticancer therapies (eg, surgery, radiotherapy).<br>• Assessing therapies for symptom management<br>• For diagnostic techniques
Line of therapy	Any	Not applicable
Manufacturer trial assessed	≥1 pivotal RCT within its report with preplanned or spontaneous crossover[a]	• Index RCT(s) with no crossover between treatment arms<br>• Index RCT(s) for which submissions do not mention crossover allowance/occurrence<br>• Clinical evidence submitted by the manufacturer is not based on an RCT (eg, non-RCTs, single-arm trials, observational evidence)<br>• Bridging studies
Outcomes	- Crossover rates<br>- Statistical significance of OS in intention-to-treat analysis	Not applicable
Publication types	Published health technology assessment reports (both single technology and multiple technology appraisals) and clinical guideline (NCCN only[b]) published by:<br>• IQWiG<br>• NICE<br>• PBAC<br>• HAS	• Submissions in progress, for which decisions have not yet been issued<br>• Documents that are not HTA submission reports (or clinical guidelines, in the case of NCCN)

Criteria	Inclusion	Exclusion
	• AEMPS<br>• AIFA<br>• CADTH<br>• ICER<br>• NCCN<br>• FDA ODAC	
Language limit	English, German, French, Spanish, Italian	Other language
Date limit	2013–current	Reports published before 2013

Abbreviations: AEMPS, Agencia Española de Medicamentos y Productos Sanitarios; AIFA, Agenzia Italiana del Farmaco; CADTH, Canadian Agency for Drugs and Technologies in Health; HAS, Haute Autorité de Santé; HTA, health technology assessment; ICER, Institute for Clinical and Economic Review; IQWiG, Institute for Quality and Efficiency in Health Care Government agency; FDA ODAC, Food and Drug Administration Oncologic Drugs Advisory Committee; NCCN, National Comprehensive Cancer Network; NICE, National Institute for Health and Care Excellence; PBAC, Pharmaceutical Benefits Advisory Committee; RCT, randomized controlled trial

[a]Crossover is defined as switching to agent(s) in the intervention arm and not switching onto a subsequent therapy which may be used as part of routine clinical practice.

[b]NCCN guidelines will be limited to the latest versions available for each of the corresponding cancer types of interest.

Table S2. HTA Search Strategy

HTA Body (URL)	HTA/Guideline Portal Navigation	Export Results	Comments
Search Approach 1: By disease (ATC classification only) or by drug			
AEMPS (https://www.aemps.gob.es)	(Translate site to English; either drop-down menu in top right of screen or use Google Translate option)<br>Medicines for Human Use > Therapeutic Positioning Reports > Search for IPTs	No export option	Google Translate option for webpage works better as the site-specific English option does not translate report titles; reports are all in Spanish, no English summary; no Boolean option. Individual cancer types cannot be searched.
AIFA (https://www.aifa.gov.it/en/)	Pricing & reimbursement > innovative medicinal products > innovative drug list (only for most recent drugs) > search in excel for drug or therapeutic area > column K for report	Assessments are listed in downloadable Excel file	67 reports in total, available in Excel
	Pricing & reimbursement > economic evaluations	No export option	Submissions between 2016 and 2022, very limited number of reports
CADTH (https://www.cadth.ca/)	Reports > Reimbursement Reviews	Results exportable into Excel (export to CSV)	Documents named differently depending on drug.
Search Approach 2: By Disease			
ICER (https://icer.org/)	Explore Our Research > Assessments > Filter by Diseases and Conditions	No export option	This site does assessments with a focus typically on drug category and not individual products; many oncology assessments are older and may be outdated.
IQWiG (https://www.iqwig.de/en/)	Projects > Projects & results > Search by keyword for disease	No export option	Many indications/drugs do not have English summaries.

HTA Body (URL)	HTA/Guideline Portal Navigation	Export Results	Comments
NCCN (https://www.nccn.org/)	Guidelines >Treatment by Cancer Type	No export option	Note that these guidelines are updated on irregular schedules and often frequently for certain cancer types; check that we have the latest version
NICE (https://www.nice.org.uk/)	Guidance > Browse guidance > Filter by title or keyword (eg, indication or treatment) Select ‘Guidance’ and ‘Technology Appraisal Guidance’ as filters (boxes on left pane of screen)	Export option: from search portal choose to view all results per page (bottom right corner of screen) and copy to clipboard (button on bottom right); this can be pasted into Excel with links intact	Boolean operators appear to work for a few terms but not for the full list of cancers; suggest testing search terms separately and then together to ensure that Boolean operators appear to be working properly
Search Approach 3: By drug name			
FDA ODAC (https://www.fda.gov/)	Google search “NAME of DRUG site:https://www.fda.gov/advisory committees”	No export option	The Google search is essentially searching the ‘site’ entered for the name of the drug. If no report is identified, for a validation step - search in Google ‘DRUG NAME FDA advisory committees’ the Google AI typically then reports whether an advisory committee meeting was conducted or not.
HAS	Use free text search function to search for topic of interest>Select Drugs and	No export option, but	English documents are summaries. Details can be found in French

HTA Body (URL)	HTA/Guideline Portal Navigation	Export Results	Comments
(https://www.has-sante.fr/jcms/pprd_2986129/en/home)	devices under “Content type” filter > drug page is stratified by indication (listed in French à need to translate) by chronological order of approval date	search can be saved and renamed	documents. Another option is to translate the web page from French to English (doable in Microsoft Edge) which would give us slightly more information than in the English PDF summary document. Note that many indications/drugs do not have English summaries.
PBAC (https://www.pbs.gov.au/pbs/home)	PBS information > Pharmaceutical Benefits Advisory Committee (PBAC) > Public Summary Documents > Public Summary Document by Product > search for treatments of interest (determine after searches of other HTA bodies completed)	No export option	Can only search by product name and view PDFs; some drugs have multiple entries which occurs if it was assessed multiple times (sometimes multiple cycles for the same indication if previously rejected, sometimes for new indications; look at most recent assessments first but all data should be collected together to understand history of submissions)

Abbreviations: AEMPS, Agencia Española de Medicamentos y Productos Sanitarios; AIFA, Agenzia Italiana del Farmaco; CADTH, Canadian Agency for Drugs and Technologies in Health; HAS, Haute Autorité de Santé; ICER, Institute for Clinical and Economic Review; IQWiG, Institute for Quality and Efficiency in Health Care Government agency; NCCN, National Comprehensive Cancer Network; NICE, National Institute for Health and Care Excellence; PBAC, Pharmaceutical Benefits Advisory Committee; pERC, pCODR Expert Review Committee; SLR, systematic literature review.

Table S3. Characteristics of 50 Included Clinical Trials

Trial Name	Disease	Crossover Rate, %	ITT OS Significance	Type of Crossover	Maturity of ITT OS	Data Cutoff	Duration of Follow-up
ALTA-1L	ALK+, mNSCLC (1L)	52.90	Nonsignificant	Built-in (protocol specified)	Immature	28 June 2019	Median follow-up of 24.9 months in the brigatinib arm vs 15.2 months in the crizotinib arm
ASCEND-4	ALK+, mNSCLC (1L)	42.70	Nonsignificant	Built-in (protocol specified)	Immature	24 June 2016	Median 19.7 months
AURA3	Metastatic/advanced EGFR and T790M mutation + NSCLC (2L)	71.0	Nonsignificant	Built-in (protocol specified - after amendment)	Mature	March 2019	Median 23.5 months for osimertinib and 20.3 months for Platinum-doublet chemotherapy
GRID	mGIST (3L)	88.00	Nonsignificant	Built-in (protocol specified)	Mature	April 2017	1708 days
KEYNOTE-177	MSI-H/dMMR mCRC (1L)	36.40	Nonsignificant	Built-in (protocol specified)	Immature	19 Feb 2020	Median duration of follow-up at the time of data cut-off was 28.4 months and 27.2 months in the pembrolizumab and SOC groups, respectively
KEYNOTE-189	EGFR–, ALK –, mNSCLC (1L)	56.30	Significant	Built-in (protocol specified)	Immature	November 2017	10.5 months

Trial Name	Disease	Crossover Rate, %	ITT OS Significance	Type of Crossover	Maturity of ITT OS	Data Cutoff	Duration of Follow-up
PROFILE1007	ALK+, mNSCLC (2L)	87.00	Nonsignificant	Built-in (protocol specified)	Mature	Not reported	Median follow-up 51.0 months in crizotinib arm and 53.1 months in the chemotherapy arm
PROFILE1014	ALK+, mNSCLC (1L)	70.00	Nonsignificant	Built-in (protocol specified)	Immature	Not reported	17.4 months in the crizotinib group and 16.7 months for those assigned to chemotherapy
RECORD-1	mRCC (2L)	81.00	Nonsignificant	Built-in (protocol specified)	Mature	November 2008	Not reported
TIVO-1	r/mRCC (1L)	62.60	Nonsignificant	Built-in (protocol specified)	Mature	Jan 2013	Median 861 days (810 for tivozanib vs 915 for sorafenib days)
VEG105192	mRCC (1L)	51.00	Nonsignificant	Natural (protocol unspecified)	Mature	March 2010	Not reported
ARCHES	mHSPC (1L)	28.80	Significant	Natural (protocol unspecified)	Immature (median NR)	28 May 2021	13.9 months
CLEOPATRA	HER2+, mBC (1L)	11.00	Significant	Natural (protocol unspecified)	Mature	February 2014	Median 49.5 months in the Perjeta group and 50.6 months in the control group

Trial Name	Disease	Crossover Rate, %	ITT OS Significance	Type of Crossover	Maturity of ITT OS	Data Cutoff	Duration of Follow-up
COU-AA-302	mCRPC, asymptomatic or mildly symptomatic (1L)	17.20	Significant	Natural (protocol unspecified)	Mature	March 2014	Median patient follow-up 49.2 months (>4 years)
DESTINY-BREAST-03	HER2+ mBC (2L+)	25.00	Significant	Natural (protocol unspecified)	Immature	May 21, 2021	A median follow-up of 16.2 months and 15.3 months, respectively, for trastuzumab déruxtécan and trastuzumab emtansine
EMILIA	HER2+, mBC (2L)	27.00	Significant	Built-in (protocol specified - after amendment)	Mature	December 2014	Median follow-up duration was 47.8 months in the Kadcyla group and 41.9 months in the capecitabine plus lapatinib group.
HER2CLIMB	HER2+, mBC (3L+)	12.90	Significant	Natural (protocol unspecified)	Mature	February 2021	Median 29.6 months
KEYNOTE-407	SQ mNSCLC (1L)	26.70	Significant	Built-in (protocol specified)	Mature	09 May 2019	Not reported
PREVAIL	mPC (1L)	4.40	Significant	Built-in (protocol specified)	Mature	March 2015	Not reported
Study301	Metastatic/advanced BC (2L)	0.40	Significant	Built-in (protocol specified)	Mature	Not reported	Not reported

Trial Name	Disease	Crossover Rate, %	ITT OS Significance	Type of Crossover	Maturity of ITT OS	Data Cutoff	Duration of Follow-up
CodeBreak-200	advanced NSCLC, KRAS-G12C mutation (2L+)	26.40	Nonsignificant	Built-in (protocol specified - after amendment)	Mature	August 2022	Median 17.71 vs 16.33 months
INVICTUS	Advanced GIST (4L+)	68.0	Significant	Built-in (protocol specified - after amendment)	Mature	January 15, 2021	Median 6.3 months primary analysis and an additional 19 months for the Jan 2021 cut-off
PROfound	mCRPC (2L+)	67.00	Significant	Built-in (protocol specified)	Mature	March 2020	21.91 months for the olaparib arm and 21.04 months for the control arm
EMPOWER-LUNG-1	PD-L1+ (>=50%) mNSCLC (1L)	42.40	Significant	Built-in (protocol specified)	Immature	March 2020	Overall ITT population: mean 14.04 (SD = 7.6) months. The overall median duration of follow-up was 13.09 months in the cemiplimab arm and 13.08 months in the chemotherapy arm.
KEYNOTE-024	PD-L1+, mNSCLC (1L)	54.30	Significant	Built-in (protocol specified)	Immature	05 January 2017	25.2 months

Trial Name	Disease	Crossover Rate, %	ITT OS Significance	Type of Crossover	Maturity of ITT OS	Data Cutoff	Duration of Follow-up
TITAN	mHSPC (1L)	39.50	Significant	Built-in (protocol specified)	Immature	September 2020	Median follow-up of final data analysis was 43.8 months
FLAURA	EGFR+ mNSCLC (1L)	41.30	Significant	Built-in (protocol specified - after amendment)	Immature	June 2019	Median follow-up of 35.8 months in the osimertinib group and 27 months in the SC group
CABOSUN	Intermediate/poor risk, locally advanced mRCC (1L)	3.80	Nonsignificant	Natural (protocol unspecified)	Mature	1 July 2017	Not reported
ALEX	ALK+ NSCLC (1L)	9.50	Nonsignificant	Natural (protocol unspecified)	Immature	February 2017	Median follow-up of primary analysis was 18.6 months
JMEN	NSQ mNSCLC (1L maintenance)	18.50	Significant	Natural (protocol unspecified)	Immature	18 December 2008	Not reported
ASCEND-5	ALK+ mNSCLC (2L)	68.10	Nonsignificant	Natural (protocol unspecified)	Mature	May 30, 2019	Median follow-up of the interim analysis was 15.87 months for the ceritinib group and 13.39 months for the chemotherapy group.

Trial Name	Disease	Crossover Rate, %	ITT OS Significance	Type of Crossover	Maturity of ITT OS	Data Cutoff	Duration of Follow-up
TROPiCS-02	HR +, HER2 – mBC (3L+)	6.70	Significant	Natural (protocol unspecified)	Mature	1 December 2022 (TROPiCS-02)	Not reported
DESTINY-Breast02	HER2+ mBC (3L+)	25.70	Significant	Natural (protocol unspecified)	Mature	30 June 2022	Median observation period for overall survival was 26.5 months in the intervention arm and 25.2 months in the control arm
CheckMate 214	Intermediate/poor risk, advanced mRCC (1L)	3.00	Significant	Natural (protocol unspecified)	Mature	7 August 2017	Median follow-up of the interim analysis was 32.72 months in the ipilimumab + nivolumab and 29.37 months in the sunitinib group
METEOR	Clear cell mRCC (2L+)	2.10	Significant	Natural (protocol unspecified)	Immature	December 2015	Minimum follow-up of 13 months
IMPACT D9901 D9902A	Asymptomatic/minimally symptomatic (non-visceral) mCRPC (1L)	67.00	Significant	Built-in (protocol specified)	Mature	Not reported	Not reported
ALUR	ALK+ NSCLC (3L)	68.60	Nonsignificant	Built-in (protocol specified)	Mature	26 Jan 2017	Median follow-up of primary analysis was 6.5 [3.5; 10.9] for alectinib and 5.8

Trial Name	Disease	Crossover Rate, %	ITT OS Significance	Type of Crossover	Maturity of ITT OS	Data Cutoff	Duration of Follow-up
							[3.8; 10.0] for the chemo group
LUX-Lung 3	EGFR+ mNSCLC (1L)	7.8-9.6	Nonsignificant	Natural (protocol unspecified)	Immature	14 November 2013	Not reported
LATITUDE	High-risk mHSPC (1L)	12.00	Significant	Built-in (protocol specified)	Mature	August 2018	Median 51.8 months
CORRECT	ECOG 0-1 mCRC (3L+)	1.57	Significant	Natural (protocol unspecified)	Mature	November 13, 2011	Not reported
CheckMate 057	NSQ mNSCLC (2L)	0.70	Significant	Natural (protocol unspecified)	Mature	2 July 2015	Minimum 17.2 months
CheckMate 017	SQ mNSCLC (2L)	4.40	Significant	Natural (protocol unspecified)	Mature	August 2015	Minimum 18 months
LAURA	EGFR+ mNSCLC (2L)	69.90	Nonsignificant	Built-in (protocol specified)	Mature	5 January 2024	Median follow-up of 22.0 months in the osimertinib group and 5.6 months in the placebo group
MAGNITUDE	BRCA 1/2 mutant mCRPC (2L+)	2.30	Significant	Natural (protocol unspecified)	Immature	15 May 2023	Median observation period: 36 months
IMpower150	NSQ mNSCLC; EGFR+/ALK+ mNSCLC (1L)	1.50	Significant	Natural (protocol unspecified)	Mature	22 Jan 2018	Not reported

Trial Name	Disease	Crossover Rate, %	ITT OS Significance	Type of Crossover	Maturity of ITT OS	Data Cutoff	Duration of Follow-up
KEYNOTE 355	mTNBC PD-L1 (CPS ≥ 10) (1L)	2.10	Significant	Natural (protocol unspecified	Mature	15 June 2021	Not reported
VISION	PSMA +, mCRPC (3L)	1.10	Significant	Natural (protocol unspecified	Mature	June 28, 2021	Not reported
PALOMA-3	HR +/HER2 - mBC (2L)	17.00	Nonsignificant	Natural (protocol unspecified	Mature	April 13, 2018	Not reported
PALOMA-2	Postemenopausal HR+/HER2- mBC (1L)	8.10	Nonsignificant	Natural (protocol unspecified	Mature	November 2021	Not reported
RELAY	EGFR+ mNSCLC (1L)	4.40	Nonsignificant	Natural (protocol unspecified	Mature	January 23, 2019	Median follow-up of 35.8 months in the osimertinib group and 27 months in the standard care group

Abbreviations: 1L, first-line; 2L, second-line; 3L, third-line; 4L+, fourth-line or further; ALK, analplastic lymphoma kinase positive; BC, Breast cancer; BRCA1/2, BReast CAncer gene 1/2; CPS, combined positive score; CRC, colorectal cancer; CRPC, castration-resistant prostate cancer; dMMR, deficient mismatch repair; ECOG, Eastern Cooperative Oncology Group; EGFR, epidermal growth factor receptor; GIST, gastrointestinal stromal tumor; HER2, human epidermal growth factor receptor 2; HR, hormone receptor; HSPC, hormone-sensitive prostate cancer; ITT, intention-to-treat; KRAS-G12C, Kirsten rat sarcoma viral oncogene homolog G12C; m, metastatic; MSI-H, microsatellite instability-high; NSCLC, non-small cell lung cancer; NSQ, non-squamous; OS, overall survival; PC, prostate cancer; PD-L1, programmed death-ligand 1; PSMA, prostate-specific membrane antigen; RCC, renal cell carcinoma; SOC, standard of care; SQ, squamous; T790M, threonine-to-methionine substitution at codon 790 of EGFR; TNBC, triple-negative breast cancer.

Figure S1. PRISMA Flow Diagram

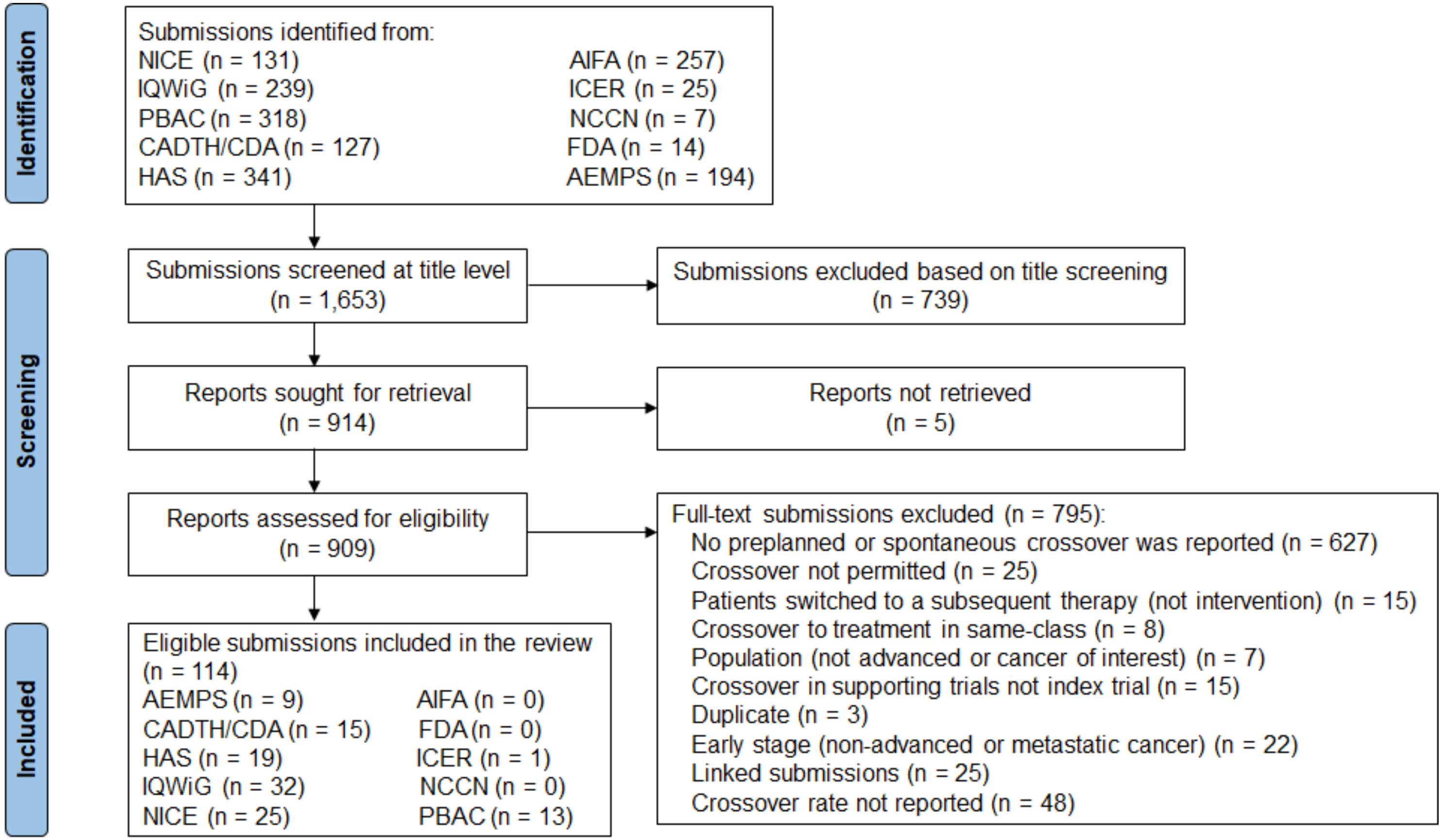

Abbreviations: AEMPS, Agencia Española de Medicamentos y Productor Sanitarios; AIFA, Agenzia Italiana del Farmaco; CADTH, Canadian Agency For Drugs And Technologies In Health; CDA, Canada's Drug Agency; FDA, Food and Drug Administration; HAS, Haute Autorité de Santé; ICER, Institute for Clinical and Economic Review; IQWiG, Institute for Quality and Efficiency in Health Care; NICE, National Institute for Health and Care Excellence; NCCN, National Comprehensive Cancer Network; PBAC, Pharmaceutical Benefits Advisory Committee; PRISMA, Preferred Reporting Items for Systematic reviews and Meta-Analyses.

Figure 2. Box Plot of the Crossover Rate by Statistical Significance Across (**A**) All Trials Regardless of Application of Crossover Mitigation Strategies, (**B**) Trials with Application of Crossover Mitigation Strategies*

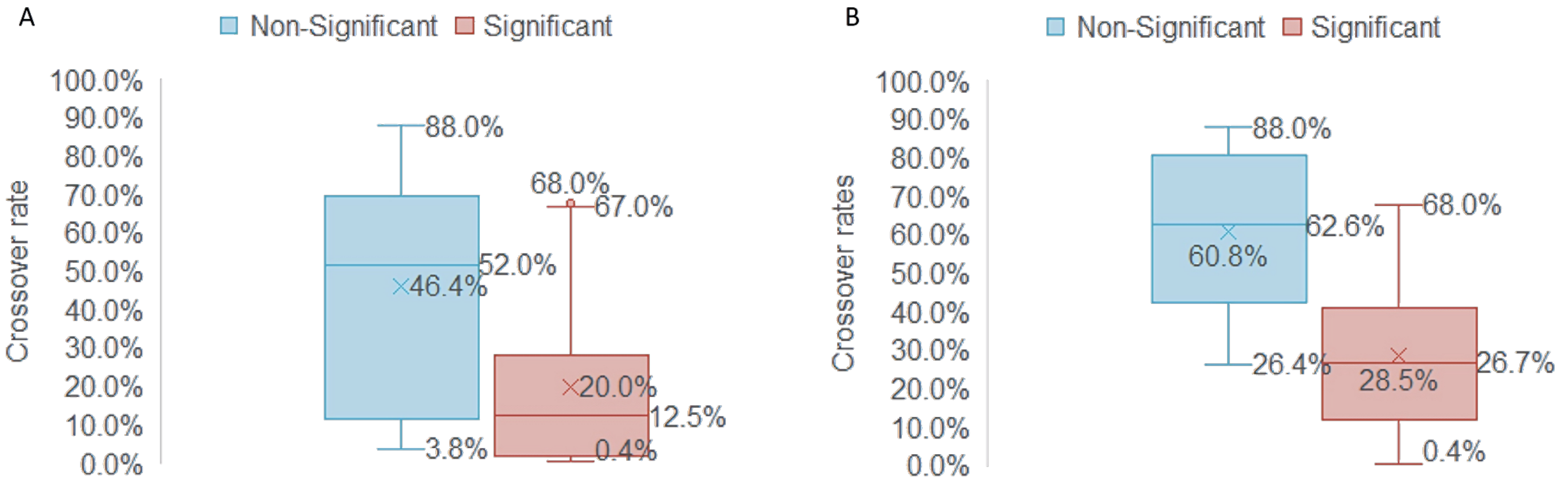

*KEYNOTE-024 and KEYNOTE-189 were excluded due to sustained OS significance at higher crossover levels driven by unusually strong early treatment effects.

Figure S3. Box Plot of the Crossover Rate by Line of Therapy Across (**A**) All Trials Regardless of Application of Crossover Mitigation Strategies, (**B**) Trials with Application of Crossover Mitigation Strategies*

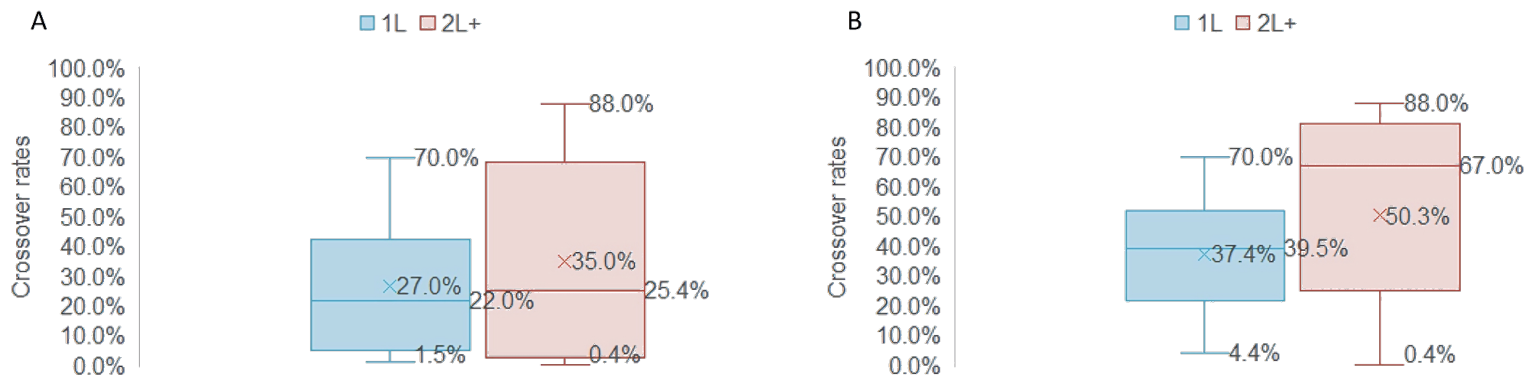

*KEYNOTE-024 and KEYNOTE-189 were excluded due to sustained OS significance at higher crossover levels driven by unusually strong early treatment effects.

Figure S4. Box Plot of the Crossover Rate by Type of Crossover Across (**A**) All Trials Regardless of Application of Crossover Mitigation Strategies, (**B**) Trials with Application of Crossover Mitigation Strategies*

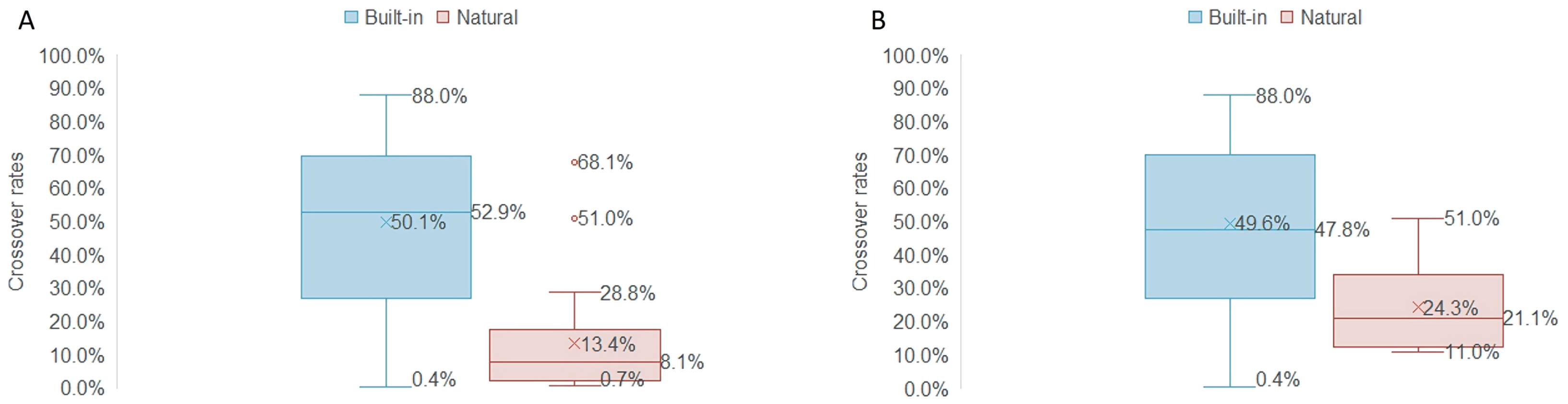

*KEYNOTE-024 and KEYNOTE-189 were excluded due to sustained OS significance at higher crossover levels driven by unusually strong early treatment effects.

Figure S5. Box Plot of the Crossover Rate by Blinding Status Across (**A**) All Trials Regardless of Application of Crossover Mitigation Strategies, (**B**) Trials with Application of Crossover Mitigation Strategies*

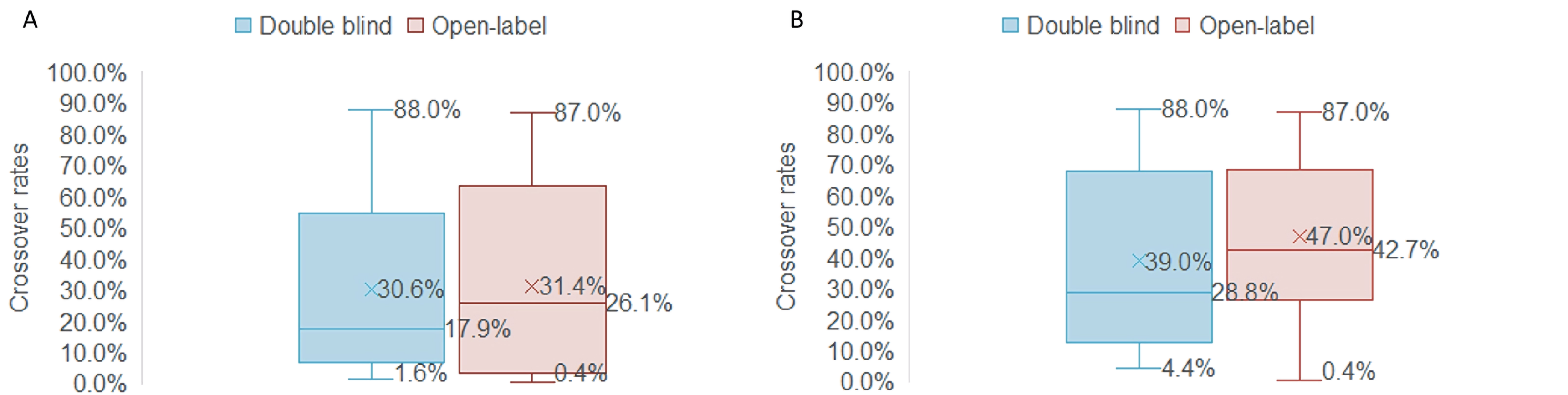

*KEYNOTE-024 and KEYNOTE-189 were excluded due to sustained OS significance at higher crossover levels driven by unusually strong early treatment effects.
